# A simple algorithm for differential diagnosis in hemodynamic shock based on left ventricle outflow tract velocity–time integral measurement: a case series

**DOI:** 10.1186/s13089-022-00286-2

**Published:** 2022-08-24

**Authors:** J. Mercadal, X. Borrat, A. Hernández, A. Denault, W. Beaubien-Souligny, D. González-Delgado, M. Vives, the Spanish Critical Care Ultrasound Network Group, Paula Carmona, David Nagore, Eduardo Sánchez, Maria Serna, Pablo Cuesta, Unai Bengoetxea, Francisco Miralles

**Affiliations:** 1 Department of Anesthesiology & Critical Care, Hospital Clinic, University of Barcelona, Barcelona, Spain; 2 Department of Anesthesiology & Perioperative Medicine, Policlinica Ibiza Hospital, Ibiza, Spain; 3 Department of Anesthesia and Intensive Care Unit, Research Centre, Montreal Heart Institute and Université de Montréal, Montreal, QC Canada; 4 Division of Nephrology, Centre Hospitalier de L’Université de Montréal, Montreal, Canada; 5 Department of Anesthesiology & Critical Care, Clínica Universidad de Navarra, Universidad de Navarra, Av. Pio XII, 36. 31008 Pamplona, Navarra Spain

**Keywords:** Transthoracic echocardiography, Cardiac output, Velocity–time integral, Hemodynamic shock, Point-of-care ultrasound, POCUS

## Abstract

Echocardiography has gained wide acceptance among intensive care physicians during the last 15 years. The lack of accredited formation, the long learning curve required and the excessive structural orientation of the present algorithms to evaluate hemodynamically unstable patients hampers its daily use in the intensive care unit. The aim of this article is to show 4 cases where the use of our simple algorithm based on VTI, was crucial. Subsequently, to explain the benefit of using the proposed algorithm with a more functional perspective, as a means for clinical decision-making. A simple algorithm based on left ventricle outflow tract velocity–time integral measurement for a functional hemodynamic monitoring on patients suffering hemodynamic shock or instability is proposed by Spanish Critical Care Ultrasound Network Group. This algorithm considers perfusion and congestion variables. Its simplicity might be useful for guiding physicians in their daily decision-making managing critically ill patients in hemodynamic shock.

## Introduction

Echocardiography is a procedure that has been used in the critical care setting for a long time. Several working groups from different societies have published guidelines and consensus documents suggesting competencies and training programs [1, 2]. However, its expansion has been limited by the absence of an accredited training. Furthermore, echocardiography is apparently complex and often requires a prolonged training in the cardiovascular imaging unit. Therefore, echocardiography is often limited to specific areas such as perioperative cardiac surgery or lung and liver transplantation.

Its use in the cardiology setting has mainly a diagnosis and prognosis role, focusing mainly in its structural perspective for medium and long-term decision-making. However, in the cardiology setting, it may also be used for functional and hemodynamic monitoring at the bed-side [3, 14–16], such as using mitral Doppler inflow patterns to guide diuresis, LVOT VTI to optimize pacing, RV systolic pressure (RVSP) estimation to guide therapy in pulmonary hypertension.

There are several algorithms aiming for guiding in the assessment of hemodynamic shock in critically ill patients, based on echocardiography [4–6]. These echocardiographic algorithms focus mainly in its structural perspective and do not offer a clear guide on how to interpret the findings in such a complex clinical context, which often leads to starting unnecessary treatment or support measures.

**Fig. 1:**
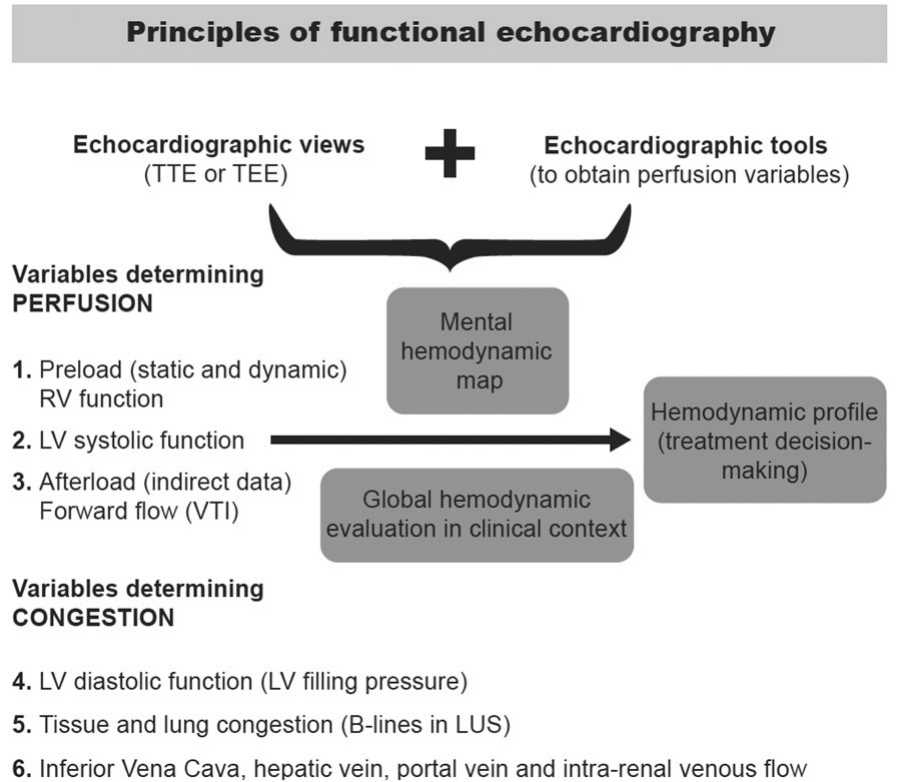
Principles of functional echocardiography

The aim of this article is to show a simple algorithm based on left ventricle outflow tract (LVOT) velocity–time integral for differential diagnosis in hemodynamic shock proposed by the Spanish Critical Care Ultrasound Network Group. This algorithm is based on LVOT velocity–time integral measurement, giving a more functional perspective to the use of echocardiography in the critical care setting. This functional perspective is based on measuring forward flow, by LVOT velocity–time integral measurement, in the LVOT or right ventricle outflow tract (RVOT), which will allow us to measure variables related to perfusion (preload, afterload and contractility). This variable integrated into clinical context may have a potential role in the differential diagnosis for hemodynamic shock in critically ill patients (Fig. 1).

Assessment of hemodynamic status is based on two concepts: perfusion and congestion. Congestion may be assessed by lung ultrasound as well as, hepatic, portal vein and venous renal congestion (VEXUS Score) along with trans-mitral inflow and E/e’ ratio to estimate LV filling pressures. However, in our article, we will focus on perfusion parameters rather than congestion.

Our review will be centered on transthoracic echocardiography, although it might also be used by transesophageal echocardiography.

Written informed consent was obtained from the patients for publication of this case report.

A case series of 4 patients is reported to whom the algorithm based on VTI was performed and its use was crucial for the differential diagnosis and management of the hemodynamic shock.

### Clinical case 1

This is a 73-year-old patient with a medical history of diabetes mellitus on insulin, hypertension and dilated ischemic heart disease (IHD) with left ventricle ejection fraction (LVEF) of 25–30% on diuretic treatment undergoing urgent cholecystectomy for acute cholecystitis. After anesthesia induction a profound hypotension (BP 60/30) and tachycardia (HR 100 bpm) refractory to volume and low-dose administration of ephedrine, is presented. It was decided to do an urgent echocardiography. From the structural perspective, a severe left ventricular systolic function impairment is observed in the echocardiography.

Step 1: What is the cause of this hemodynamic shock? According to clinical context and medical history it could be the following: (a) septic shock due to cholecystitis, or (b) cardiogenic shock secondary to an acute ischemic cardiomyopathy, or (c) relative hypovolemia due to diuretics.

Step 2: What treatment would you give? (1) Fluid therapy, with its associated risk of overloading; (2) vasopressors, with its associated risk of lowering cardiac output and worsening hypoperfusion; (3) inotropic drugs, with its associated risk of worsening the clinical state if hypovolemia or IHD is the cause of the shock.

According to structural algorithms, the images showing low LVEF in the TTE would lead to initiation of inotropic support to improve contractility, whereas with the use of echocardiography from the hemodynamic and functional perspective, an adequate forward flow (VTI of 19 cm and cardiac output of 5,9L/min) is shown. As it is a chronic dilated cardiomyopathy with high end-diastolic volumes, an adequate stroke volume was maintained. Inferior vena cava (IVC) diameter was 20 mm with a distensibility of 10%. Mitral-inflow E/A ratio was 1.2.

The VTI of 19 cm, calculated by TTE, helped us in our differential diagnosis, leading us to suggest that the most likely cause of hemodynamic shock was the low vascular resistance due to anesthetic drugs and treating with vasopressors rather than inotropic support or fluids.

### Clinical case 2

A 67-year-old male patient was admitted to ICU for a post-operative shock state after surgery. As a medical history, he has chronic kidney disease on hemodialysis, two previous episodes of Pulmonary Embolism (PE) on anticoagulant treatment and chronic IHD secondary to a myocardial infarction (MI) in 2006. After reversal of anticoagulation, he underwent surgery for an arteriovenous peripheral abscess drainage in the left arm. Intraoperatively, there was significant bleeding, estimated as 1 L, due to extension of abscess to deep planes. Two-hours after surgery severe hypotension with tachycardia of 110 bpm and signs of tissular hypoperfusion (lactates of 4.5 mmol/L and ScvO2 63%) occurred.

Step 1: What is the cause of this hemodynamic shock? According to clinical context and medical history it could be the following: (a) hypovolemia secondary to bleeding, (b) septic shock due to abscess, or (c) obstructive shock due to a potential new PE or (d) cardiogenic shock secondary to a new MI.

Step 2: What treatment would you give? (1) Fluid therapy, with its associated risk of overloading as he is patient on hemodialysis; (2) vasopressors, with its associated risk of lowering cardiac output and worsening hypoperfusion; (3) inotropic drugs, with its associated risk of worsening the clinical state if hypovolemia or vasoplegia is the cause of hemodynamic shock.

LV systolic function, observed in the 4-chamber apical view, is estimated to be moderately impaired (LVEF estimated to be around 35%). RV systolic function assessment includes a RV/LV area ratio of 0.9 and a TAPSE of 13 mm measured by M-mode, suggesting a moderate RV systolic dysfunction and mild RV dilatation.

SV is measured by normalized VTI, and a VTI of 21 cm is measured at the LVOT level in the 5-chamber apical view. Fluid responsiveness is assessed by a PLRT and VTI is increased by 13% (from 21 to 24 cm), suggesting that patient is fluid responsive. IVC diameter was 15 mm and distensibility of 20%.

As a summary, this is a patient with a moderately impaired LV systolic function, as well as, a moderately impaired RV systolic function, who is fluid responsive, has a stroke volume of 66 mL estimated by a normalized VTI.

Almost all echocardiographic structural parameters related to perfusion are impaired. Consequently, these values may be confusing.

What echocardiographic perfusion parameters should be prioritized to determine the main contributor to this hemodynamic shock? How to distinguish between chronic or acute impaired perfusion parameters?

Following our algorithm, first step is to determine whether an adequate forward flow is present or not. In this clinical case VTI at LVOT is 21 cm with a HR of 110 bpm, and CO is 7.2L/min. As a result, an adequate SV or forward flow is suggested. Therefore, in the context of hemodynamic shock associated with hypotension, the main contributor is a distributive shock secondary to low systemic vascular resistance (SVR). However, the patient is also fluid responsive with signs of tissular hypoperfusion (lactate 4.5 mmol/L and ScvO2 63%). Therefore, fluid therapy was given first, followed by vasopressors with significant hemodynamics improvement and re-assessment was performed later.

### Clinical case 3

A 42-year-old women, with a perforated duodenal ulcer secondary to NSAIDs required an urgent laparotomy complicated with bleeding. Diffuse secondary peritonitis and severe hemodynamic shock, requiring high dose of noradrenaline (0.4mcg/kg/min), developed postoperatively. She had no past medical history. Biomarkers of infection were increased with a procalcitonin of 20 ng/mL and C-reactive protein of 30 mg/l.

Step 1: What is the cause of this hemodynamic shock? According to clinical context and medical history it could be the following: (a) hypovolemia secondary to bleeding, (b) septic shock due to secondary peritonitis, or (c) obstructive shock due to a PE or (d) cardiogenic shock secondary to a stress cardiomyopathy or a new MI.

Step 2: What treatment would you give? (1) Fluid therapy, with its associated risk of overloading; (2) vasopressors, with its associated risk of lowering cardiac output and worsening hypoperfusion; (3) inotropic drugs, with its associated risk of worsening the clinical state if hypovolemia or vasoplegia is the cause of hemodynamic shock.

24 h postoperatively, an inferolateral elevated ST was observed in ECG. LV global and regional contractility was not impaired and LVOT VTI was preserved (21,5 cm). Few hours later, bigeminy was observed. An anterior, anteroseptal medial and apical akinesia, with a VTI of 14 cm in LVOT and LVEF of 35% was shown in TTE. IVC was 22 mm and distensibility with mechanical ventilation was 5%. Trans-mitral inflow E/A ratio was 1.9. Hypotension and tachycardia (100 bpm) were developed requiring increasing dose of vasopressors and adding vasopressin at 0,03UI/min.

The low VTI of 14 cm associated with a dilated IVC with no distensibility and E/A ratio of almost 2, suggested that the cause of the hemodynamic shock was a mixed distributive and cardiogenic shock due to stress cardiomyopathy, confirmed by a normal coronary angiography.

Dobutamine infusion was initiated at 5 mcg/kg/min and diuretics were given with a significant hemodynamic improvement leading to a decrease of noradrenaline and increase of VTI in LVOT to 17 cm. Dobutamine was stopped 4 days later because LV contractility had recovered and VTI at the LVOT improved (21 cm).

### Clinical case 4

A 65-year-old man with a perforated ascending colon due to a cancer requiring a right colectomy by an urgent laparotomy. Subsequently, a diffuse secondary peritonitis and a hemodynamic shock, requiring high dose of vasopressors (noradrenaline at 0,3mcg/kg/min), was developed. As a medical history, he had hypertension, diabetes, and ischemic cardiomyopathy with preserved LVEF. During surgery bleeding of 1.5 L occurred.

24 h postoperatively, hypotension and tachycardia were progressively increasing requiring increasing dose of vasopressors up to 0,5mcg/kg/min and adding vasopressin at 0,03UI/min, with signs of tissular hypoperfusion (lactate 3,5 mmol/L and ScvO2 64% and oliguria < 0,5 ml/kg/h).

Step 1: What is the cause of this hemodynamic shock? According to clinical context and medical history it could be the following: (a) hypovolemia secondary to bleeding, (b) septic shock due to secondary peritonitis, or (c) obstructive shock due to a PE or (d) cardiogenic shock secondary to a stress cardiomyopathy or a new MI.

Step 2: What treatment would you give? (1) Fluid therapy, with its associated risk of overloading; (2) vasopressors, with its associated risk of lowering cardiac output and worsening hypoperfusion; (3) inotropic drugs, with its associated risk of worsening the clinical state if hypovolemia or vasoplegia is the cause of hemodynamic shock.

TTE showed a not dilated LV with a moderate inferoseptal and inferior hypokinesia. RV was not dilated, and contractility was not impaired. IVC had a diameter of 8 mm with a distensibility of 20%. VTI at LVOT was 16 cm. Trans-mitral inflow E/A ratio was 0.9 suggesting low LV filling pressures. A passive leg raising test was performed and a VTI increased of 15% occurred, suggesting that the patient could benefit from fluid therapy. Hemodynamics improved with the administration of 500 cc of fluid therapy allowing us to decrease noradrenaline dose down to 0,25mcg/kg/min.

The low VTI (16 cm) associated with a small IVC of 8 mm with a distensibility of 20%, along with, low filling pressures with E/A ratio 0.9 and fluid responsive after a PLRT, suggested that the patient was hypovolemic and not yet well filled intravascularly (Table 1).

**Table 1:** Patients’ characteristics of the case series to whom our algorithm based on VTI was performed

Patient	Clinical scenario	VTI and others TTE parameters	Cause of hemodynamic shock	Therapeutic strategy
1	- Anesthesia induction for cholecystectomy due to acute cholecystitis -IHD with LVEF 25%	- LVOT VTI 19 cm - IVC of 20 mm and distensibility of 10% - Mitral-inflow E/A ratio 1.2	- Distributive Shock secondary to anesthetic drugs	Vasopressors (phenylephrine or noradrenaline)
2	- Postoperative of an arteriovenous peripheral abscess drainage complicated with bleeding requiring polytransfusion - CKD on IHD - Previous PE. IHD	- LVOT VTI 21 cm - Fluid responsive - IVC diameter 15 mm and distensibility of 20%	- Distributive shock secondary to either SIRS or Sepsis 2ry to the abscess. Along with, being fluid responsive with signs of tissular hypoperfusion	Fluid therapy first followed by vasopressors
3	- Postoperative of a diffuse secondary peritonitis secondary to perforated duodenal ulcer complicated with bleeding -No past medical history	- LVOT VTI 14 cm - IVC was 22 mm and distensibility was 5% - Mitral-inflow E/A ratio 1.9	- Mixed Shock (distributive plus cardiogenic) due to a stress cardiomyopathy	Dobutamine infusion and diuretics
4	- Postoperative of a diffuse secondary peritonitis 2ry to a perforated ascending colon due to a cancer requiring a right colectomy complicated with severe bleeding - Hypertension - Diabetes - IHD with preserved LVEF	- LVOT VTI 16 cm - IVC 8 mm with a distensibility of 20% - Mitral E/A ratio 0,9 - Fluid responsive with a 15% increase in LVOT VTI after a PLRT	- Hypovolemic shock due to severe bleeding during surgery with signs of tissular hypoperfusion	- Fluid therapy - Maintain or decrease vasopressors if possible

## Discussion

In this article, a case series of patients have been reported to whom the algorithm based on LVOT velocity–time integral measurement was performed, and its use was crucial for differential diagnosis and management of hemodynamic shock.

For the assessment of perfusion, it is essential to measure the CO. CO calculation often justifies the use of invasive monitors such as the pulmonary artery catheter pulmonary (PAC) or other transpulmonary thermodilution-based systems.

CO calculation by echocardiography non-invasively has been compared to thermodilution by the pulmonary artery catheter (PAC), showing an excellent performance. Data from a prospective cohort study on 50 cardiac surgery patients determining the correlation between ultrasound (US) and PAC, showed an excellent performance to detect a 10% variation in CO (Receiver operating curve (ROC 0.9; specificity 71% and sensitivity 92%) [7]. More recently, data from a meta-analysis on 1,996 patients from 68 studies, no significant differences were found between US and thermodilution-derived CO (mean difference − 0.14; 95% CI − 0.30 to 0.02; *p* = 0.08) [8].

Cardiac ultrasound allows calculating SV by using pulsed wave Doppler (PWD) technique. PWD calculates the velocity of red blood cells at a specific point in the cardiovascular system. If PWD is applied at the level of the LVOT, a triangular image will be obtained that represents the spectrum of velocities of the red blood cells that pass through this point during each beat.

**Fig. 2:**
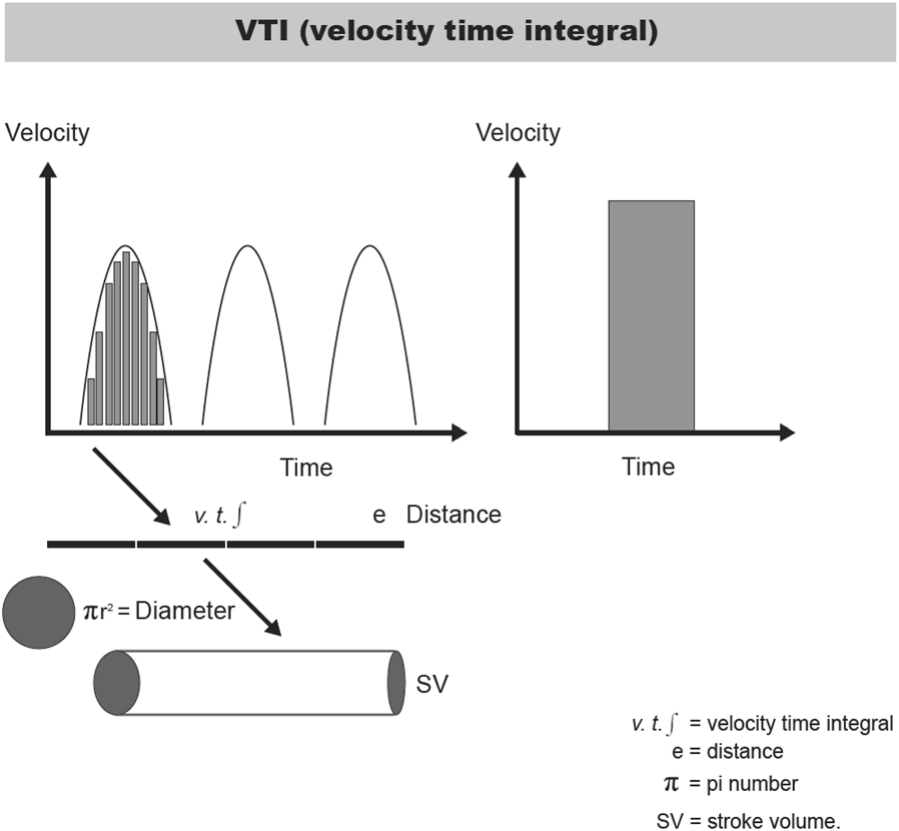
Mathematical explanation for VTI calculation

If the surface of such a triangle is traced with our ultrasound system, the VTI, will be obtained, which is expressed in cm (Fig. 2).

VTI is the length of the column of blood passing through a single point in each heartbeat. It is considered that the LVOT is a circular structure that does not significantly change its shape during the cardiac cycle.

The volume of blood ejected from the left ventricle during systole passes through the LVOT, which is shaped like a cylinder. Solving for the volume of that cylinder, therefore, yields the SV (Fig. 2):

$${\text{Cylinder volume}}\, = \,{\text{height }} \times {\text{ CSA,}}$$

where CSA indicates the cross-sectional area. The height of the cylinder is the LVOT VTI, obtained by pulsed wave (PW) Doppler placed just proximal to the aortic valve in an apical five- or three-chamber view.

The SV may be calculated by multiplying VTI by the cross-section area (CSA) at the level of LVOT: CSA = Π × (diameter LVOT/2) [2]. The main limitation with this measurement is that calculation of LVOT diameter (LVOTd) is a measurement with poor reproducibility. Consequently, when interpreting the progression of the CO, it is unknown whether the changes are related to treatment or differences in the LVOTd measurement. A well acquired LVOT VTI is represented by a spectral envelope, which is “dark” on the inside with a bright “outline.” LVOT VTI should include the closure "click" of the aortic valve in the Doppler profile.

It was chosen to convert the LVOT in a constant parameter, given that, it is a value that does not change significantly over time and is proportional to body surface area (BSA) [9]. This simplification allows us to assume that SV = VTI. Therefore, the changes in VTI reflect changes in the SV. This has already been mentioned in a consensus document on monitoring in hemodynamic shock by a working group from the ESICM [10].

In this way, it is possible to estimate SV by normalizing the LVOTd to the global population mean, LVOTd of 2 cm (20.3 ± 2.3 mm) [9, 11–13].

If the LVOTd is normalized, assuming the global population mean, the following formula may be used: SV = VTI × [Π × (2/2)^2^] = VTI × Π (= 3.14).

**Fig. 3:**
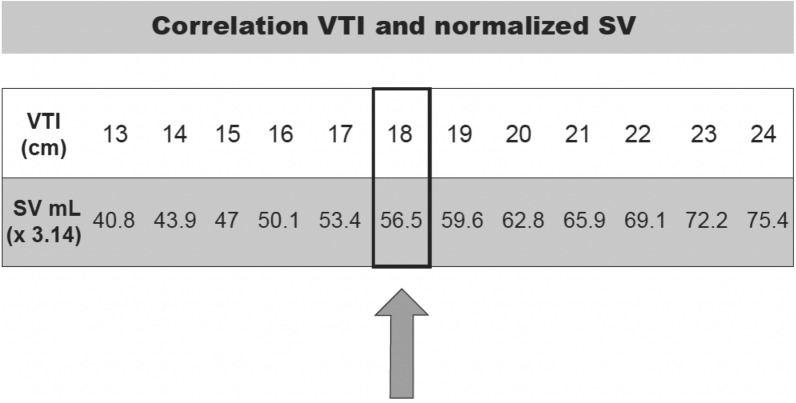
Correlation of VTI and normalized SV

Based on this formula, SV may be calculated based on VTI values. A calculated VTI of 18 cm, corresponds to an SV of 56.5 mL, which is very close to 60 mL, which is the lower limit of normality, according to hemodynamic literature and the cut-off value to define a low SV (Fig. 3).

A VTI of 18 cm has been used as the cut-off value for low SV for several reasons. First, it is assumed that there is a tendency to underestimate the VTI, due to a suboptimal Doppler bean alignment between our beam and the orientation of the LVOT. Thus, it is possible to assess perfusion using VTI directly, either in the LVOT or RVOT [12–14]. Second, guidelines from the American Society of Echocardiography (ASE) for the echocardiography as a monitor for therapeutic intervention in adults suggest VTI > 18 cm, as an adequate CO [15]. This suggestion is based on data from an observational cohort study on 990 ambulatory patients with stable coronary artery disease (CAD), where it is observed that a VTI < 18 cm is associated with an increase rate of heart failure requiring hospitalization and mortality independent of clinical and other echocardiographic parameters [16].

A recent editorial was emphasizing and suggesting that hemodynamic monitoring is best achieved with the use of a simple quantitative measurement reflecting stroke volume—VTI at the LVOT or RVOT—and that this measurement should be taught to every operator at the earliest stage of echo training. The author suggested that since flow is such a crucial indicator of tissue perfusion and the target of most of our therapeutic interventions, it seems reasonable to include LVOT VTI measurement in the curriculum of “basic” echo training [17]. This approach has also been supported by other experts in the field of critical care echocardiography [18]. The view to be used for LVOT VTI is apical 5-chamber or apical 3-chamber view. PWD at the LVOT, 1 cm proximal to the aortic valve, should be used to measure VTI. As already mentioned above, the value of 18 cm is the minimum normal value of VTI, which discriminates between normal or low SV.

The average overall RVOT distal diameter is slightly larger than LVOTd (21.7 ± 3.14 mm vs 20.3 ± 2.3 mm) [13]. Therefore, the RVOT VTI cut-off value should be slightly lower (from 18 to 15 cm) than LVOT VTI. The view to be used for RVOT VTI is subcostal short-axis RVOT or parasternal long-axis RV outflow. This value as the cut-off value should be interpreted into clinical context. Critical care setting is complex, but for the sake of simplicity it is useful to have a clear figure in memory to serve as a reference.

The main limitations for using VTI as SV calculation are the following:

1. SV calculation will be overestimated if the following pathologies are present: (a) subaortic stenosis, (b) LVOT dynamic obstruction associated with systolic anterior motion (SAM), (c) moderate-to-severe aortic regurgitation, and (d) the presence of aortic prosthetic valve. To overcome this limitation, measurement of VTI at the RVOT, placing the PWD just before the pulmonary valve, by the subcostal short-axis RVOT or parasternal long-axis RV outflow, may be used.
2. Either a smaller (SV will be overestimated) or larger (SV will be underestimated) LVOT diameter than mean global population in people with low or high body surface area (BSA). To overcome this limitation, the LVOTd should be measured by an expert sonographer.

### VTI-based algorithm in hemodynamic shock proposal

**Fig. 4:**
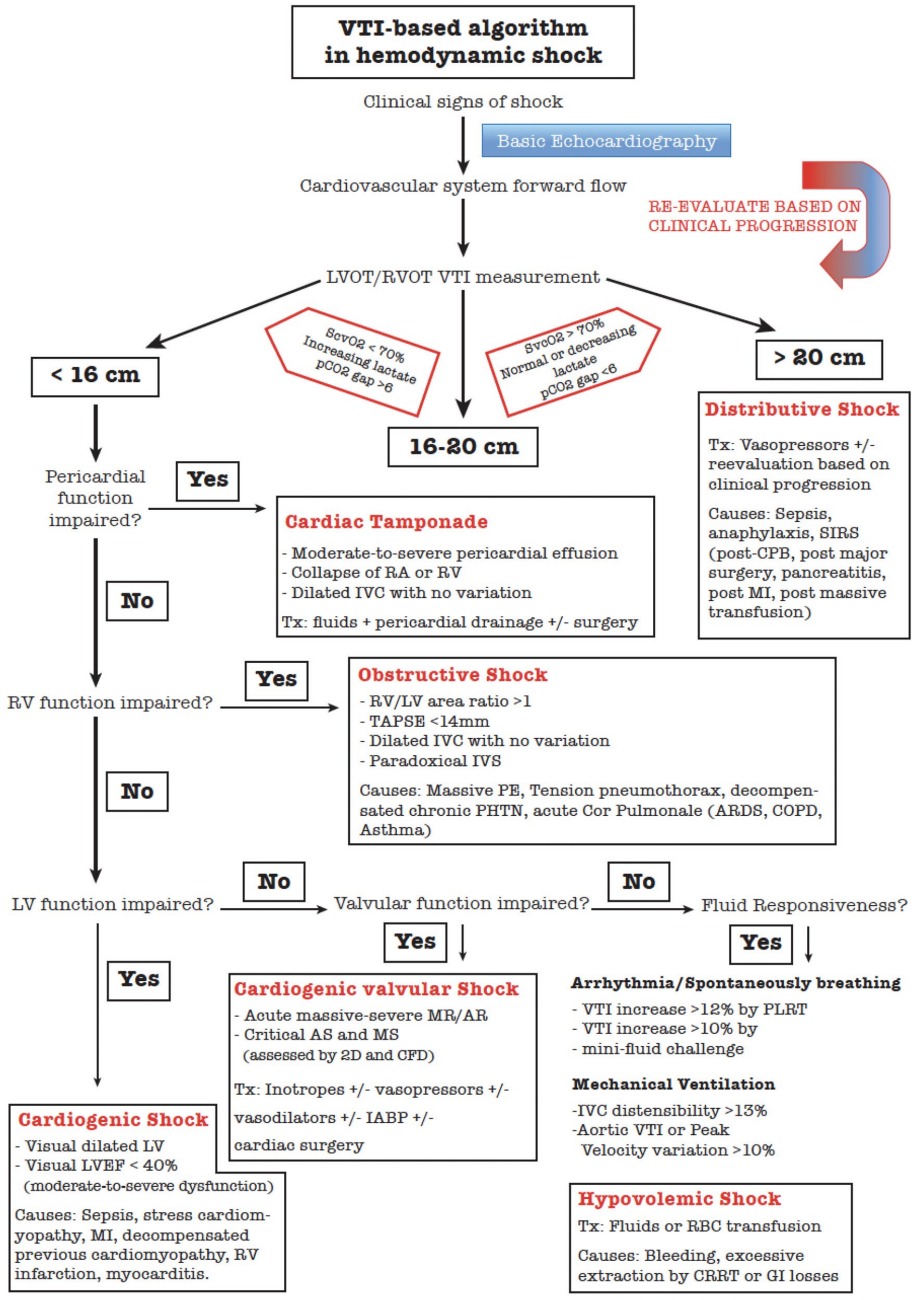
VTI-based algorithm in hemodynamic shock. *AR* aortic regurgitation. AS, aortic stenosis, *ARDS* acute respiratory distress syndrome, *CFD* color flow Doppler. *COPD* chronic obstructive pulmonary disease, *CRRT* continuous renal replacement therapy, *GI* gastrointestinal, *MI* myocardial infarction, *IABP* intra-aortic balloon pump, *IVC* inferior vena cava, *IVS* intraventricular septum, *LV* left ventricle, *LVEF* left ventricle ejection fraction, *LVOT* left ventricle outflow obstruction. *MS* mitral stenosis, *PE* pulmonary embolism, *PLRT* passive leg raising test, *PHTN* pulmonary hypertension, *RA* right atrium, *RBC* red blood cells, *RV* right ventricle, *RVOT* right ventricle outflow tract, *TAPSE* tricuspid annular plane systolic excursion, *VTI* velocity–time integral

In this article, an algorithm based on LVOT velocity–time integral measurement it is proposed by the Spanish Critical Care Ultrasound Network Group, for hemodynamic shock assessment, guiding in the decision-making for differential diagnosis (Fig. 4).

First step is to analyze whether the patient has an adequate forward flow in terms of SV and CO. This first step will be answered by the measurement of the normalized VTI at LVOT or RVOT:

- 1. If VTI is above 20 cm, the SV is adequate and therefore the cause of the hemodynamic shock will most likely be a distributive shock. In this case, after initiating vasopressors to normalized mean blood pressure, VTI will be reassessed to ensure SV has not been reduced by increasing afterload related to vasopressors.
- 2. On the other hand, if VTI is below 16 cm, an abnormally low SV is suggested, and the cause of this low-cardiac output syndrome should be diagnosed. The lower limit of VTI has been downgraded to 16 cm because most of the time in critical care, images obtained are less than optimal and there is a tendency to underestimate the VTI, due to a suboptimal Doppler bean alignment. Therefore, VTI between 18 ± 2 is left as a “gray-zone” where clinical assessment is of paramount importance.
- 2.1First, pericardial function is evaluated to rule out cardiac tamponade.
- 2.2 Second, RV systolic function and size will be evaluated. If RV systolic function is impaired or severe RV dilatation is present, the most likely cause of the hemodynamic shock is obstructive shock, e.g., massive pulmonary embolism (PE), and it may be treated accordingly. However, differential diagnosis includes other causes of RV pressure or volume overload, such as acute cor pulmonale (ARDS, COPD, asthma), tension pneumothorax, RV infarction, decompensated chronic pulmonary hypertension (PHTN), fluid overload or stress-related RV systolic impairment (sepsis cardiomyopathy).
- 2.3Third, if RV systolic function is normal, LV systolic contractility will be assessed by eyeballing. LV filling pressure will be measured by mitral-inflow E/A ratio. If LV systolic function is moderate or severely impaired, in absence of hypovolemia, the most likely cause is cardiogenic shock, including diastolic dysfunction, sepsis stress-cardiomyopathy, MI, decompensated previous cardiomyopathy, myocarditis.
- 2.4Fourth, if massive mitral or aortic regurgitation is observed, it could be a main contributor of the hemodynamic shock.
- 2.5Lastly, fluid responsiveness will be assessed. If patient is responsive, fluid therapy will be given.

The order followed for the perfusion parameters assessment in our algorithm is not trivial. The goal is to recover perfusion parameters without over resuscitating the patient avoiding adverse effects. For this reason, fluid responsiveness is the last one to be evaluated, after ruling out tamponade, RV/LV systolic function and left-sided valvular incompetency.

To achieve this goal, our premise is the following: a structural cardiac dysfunction does not significantly contribute to the hemodynamic shock, unless causes a low-cardiac output syndrome, measured by a normalized VTI below 16 cm. Therefore, it should not be treated. Even if the patient is fluid responsive and VTI is normal (VTI > 18 cm), fluid therapy may not be recommended, unless signs of tissular hypoperfusion are present. This is the normal physiological state and if SV is not low, fluid therapy may lead to worse outcomes associated with adverse effects secondary to overloading [19–21].

The presence of regional or global wall motion abnormalities does not necessarily mean it should be treated with inotropic support unless low SV (VTI < 18 cm) occurs. Inotropic support is indicated for low-cardiac output syndrome associated with signs of tissular hypoperfusion unless you have LV or RV outflow obstruction [10].

The use of inotropic support in a patient with an adequate SV (VTI > 18 cm) might lead to an increased myocardial mechanical stress and oxygen consumption with its risk of arrhythmias.

The presence of RV contractility impairment or RV dilatation associated with an adequate SV (VTI > 18 cm) means that the RV dysfunction is not the cause of hypotension or hemodynamic shock. As an example, if a patient is diagnosed of PE in the clinical context of hemodynamic shock with normal SV, the RV dysfunction will, most likely, not be the main cause of shock. It would rather be a distributive shock, requiring to rule out septic shock and controlling its source.

Overall, if there is no benefit from using a specific treatment, it should be avoided to decrease its associated adverse effects.

Our algorithm is not intended to be an algorithm that fits all possible clinical scenarios, since it would be too long and therefore not useful at the clinical level. Tension pneumothorax and LVOT/RVOT obstruction would be fluid responsive. However, they are out of the scope of our algorithm. Combined RV and LV systolic impairment would include both clinical scenarios. Abdominal compartment syndrome would not be included in our algorithm.

The measurement of LV filling pressure by mitral-inflow E/A ratio > 2 could only be used when LV systolic function impairment exists. Furthermore, only E/A > 2 and E/A < 0.8 + E velocity < 0.5 m/s can be used in isolation to clearly define LV filling pressure in those patients. The range of E/A 0.8—2.0 is indeterminate and required to measure additional variables (left atrium size, E/E’ ratio and tricuspid regurgitation peak velocity). Also, LV filling pressure can be normal with an E/A > 2 in patients with normal diastolic function (young < 40 years old). The criteria to accurately assess fluid responsiveness by aortic valve peak velocity or VTI variation associated with invasive mechanical ventilation are well known: (a) sinus rhythm with no premature atrial or ventricle contraction; (b) tidal volume > 7 ml/kg; (c) absence of inspiratory efforts and lastly, but more importantly, absence of RV systolic dysfunction.

IVC distensibility test requires the following criteria to perform well: (a) tidal volume >  = 8 ml/kg; (b) PEEP no more than 5; (c) absence of inspiratory efforts or assisted pressure support; (d) absence of moderate-to-severe tricuspid regurgitation or RV dysfunction; (e) absence of intra-abdominal hypertension and, lastly, absence of cardiac tamponade.

It is proposed as a mental guide to prioritize concepts and facilitate the integration of echocardiography into patient’s clinical context for clinical decision-making.

To conclude, several topics will be discussed for the algorithm to perform well:

- Once the treatment has been applied, echocardiographic re-assessment to confirm SV improvement should be performed. Reassessment is also crucial when a significant hemodynamic change occurs (e.g., tachycardia, increase need for vasopressors/inotropes and hypoperfusion refractory to treatment).
- Clinical situations where VTI is in the “gray-zone” (VTI between 16 and 20), it is crucial the clinical context and tissue perfusion assessment by measurement of arterial lactate, central venous oxygen saturation, CO2 venous–arterial gradient. If hypoperfusion persist, despite having optimized oxygen arterial saturation and hemoglobin, measures to increase SV should be applied. The “gray-zone” of VTI between 16 and 20 cm could be debated. Sattin et al. [22] have recently suggested a systematic approach for using SV measurements to help integrate important 2D findings into the clinical context. They proposed a “gray-zone” of VTI between 14 and 22 cm, suggesting to measure the LVOTd in case of a VTI in the “gray-zone” between 14 and 22 cm. However, LVOTd measurement has poor reproducibility. Any small error in measuring the LVOTd may lead to a significant overestimation or underestimation of the SV. Therefore, we suggest that only experienced sonographers perform LVOTd measurement. The average overall RVOT distal diameter is slightly larger than LVOTd (21.7 ± 3.14 mm vs 20.3 ± 2.3 mm) [13]. Therefore, the RVOT VTI cut-off value should be slightly lower (from 18 to 15 cm) than LVOT VTI.
- If tachycardia is present, it should be evaluated whether it is a compensatory or a reactive tachycardia. Compensatory tachycardia is secondary to a low CO syndrome (e.g., cardiogenic, obstructive, or hypovolemic shock), whereas a reactive tachycardia is secondary to an inflammatory state such as distributive shock (e.g., Sepsis or Systemic Inflammatory Response Syndrome—SIRS), associated with a normal or high CI > 2.5 L/min/m^2^. If CI is < 2.2 L/min/m^2^, compensatory tachycardia is suggested and treatment to increase SV either with fluids or inotropes, should be applied.
- If AF is present, the mean of five VTI measures should be used for a normalized VTI and CI calculation.

## Conclusion

In this article, a case series of patients have been reported to whom the algorithm based on LVOT velocity–time integral measurement was performed and its use was crucial for differential diagnosis and management of hemodynamic shock.

This simple algorithm aims for guiding in the decision-making for the differential diagnosis of hemodynamic shock. It represents a shift in the classic hemodynamic shock assessment and management guide by echocardiography. This shift consists of moving from a more structural perspective to a more functional one. Our functional perspective aims for integrating all perfusion parameters through a simple algorithm, rather than evaluating structural abnormalities separately, with no integration into a functional assessment. Ultimately, it is a useful tool for individualizing treatment, detecting the main contributor to the hemodynamic shock state, followed by an early appropriate treatment, avoiding fluid overloading or inotropes overuse. Whether the use of this simple algorithm VTI-based in hemodynamic shock may improve important clinical outcomes remains to be elucidated.

## Data Availability

The datasets used and/or analyzed during the current study are available from the corresponding author on reasonable request.

## Abbreviations

AF: Atrial fibrillation
AMI: Acute myocardial infarction
ARDS: Acute respiratory distress syndrome
ASE: American Society of Echocardiography
BP: Blood pressure
BSA: Body surface area
CAD: Coronary artery disease
CI: Cardiac index
CO: Cardiac output
CSA: Cross-sectional area
CVP: Central venous pressure
HR: Heart rate
IVC: Inferior vena cava
LVEF: Left ventricle ejection fraction
LVOT: Left ventricle outflow tract
MAP: Mean arterial pressure
P1: Pressure 1
P2: Pressure 2
PAC: Pulmonary artery catheter
PE: Pulmonary embolism
PLRT: Passive leg raising test
PWD: Pulse wave Doppler
RVOT: Right ventricle outflow tract
SAM: Systolic anterior motion
SIRS: Systemic inflammatory response syndrome
SV: Stroke volume
SVR: Systemic vascular resistance
TEE: Transesophageal echocardiography
TTE: Transthoracic echocardiography
US: Ultrasound
VTI: Velocity–time integral
